# Impact of Tyrosine Kinase Inhibitors (TKIs) Combined With Radiation Therapy for the Management of Brain Metastases From Renal Cell Carcinoma

**DOI:** 10.3389/fonc.2020.01246

**Published:** 2020-07-23

**Authors:** Muhammad Khan, Zhihong Zhao, Sumbal Arooj, Guixiang Liao

**Affiliations:** ^1^Department of Radiation Oncology, Shenzhen People's Hospital, The First Affiliated Hospital of Southern University of Science and Technology, Shenzhen, China; ^2^Department of Oncology, First Affiliated Hospital of Anhui Medical University, Hefei, China; ^3^Department of Nephrology, Shenzhen People's Hospital, Second Clinical Medicine Centre, Jinan University, Shenzhen, China; ^4^Department of Biochemistry, University of Sialkot, Sialkot, Pakistan

**Keywords:** tyrosine kinase inhibitors (TKI), brain metastases (BM), metastatic renal cell carcinoma (mRCC), overall survival (OS), brain control (BC), stereotactic radiosurgery (SRS)

## Abstract

**Background:** Targeted therapy has transformed the outcome for patients with metastatic renal cell carcinoma. Their efficacy and safety have also been demonstrated in brain metastatic RCC. Preclinical evidence suggests synergism of radiation and tyrosine kinase inhibitors. Consequently, several studies have compared their efficacy in the treatment of RCC brain metastases to the era of brain management with surgery/radiation only.

**Objectives:** We seek to systematically review and meta-analyze the results of those studies that involved comparative intervention groups of brain management; TKIs, and never used TKIs.

**Methods and Materials:** Online databases (PubMed, EMBASE, Cochrane library, and ClinicalTrials.gov) were searched for comparative studies. Overall survival as the primary outcome of interest, and local brain control, distant control, and adverse events as secondary outcomes of interest were recorded for meta-analysis. Hazard ratios were pooled together using Review Manager 5.3. Fixed effects or random effects model were adopted according to the level of heterogeneity. Subgroup analysis included studies that involved SRS as the local treatment of management.

**Results:** Overall 7 studies (*n* = 897) were included for meta-analysis. TKI use was associated with better survival (HR 0.60 [0.52, 0.69], *p* < 0.00001) and local brain control (HR 0.34 [0.11, 0.98], *p* = 0.05). SRS subgroup also revealed significantly better survival (HR 0.61 [0.44, 0.83], *p* = 0.002) and local brain control (HR 0.19 [0.08, 0.45], *p* = 0.0002). Distant brain control (HR 0.95 [0.67, 1.35], *p* = 0.79) and brain progression free survival were unaffected (HR 0.94 [0.56, 1.56], *p* = 0.80). Only one study (*n* = 376) reported significantly greater 12-months cumulative incidence of radiation necrosis with TKI use within 30 days of SRS (10.9 vs. 6.4%, *p* = 0.04).

**Conclusions:** TKIs use in combination with SRS is safe and effective for treating RCC brain metastases. Larger randomized controlled trials are warranted to validate the results.

## Introduction

Renal cancer is the eighth leading cancer type according to estimated new cancer cases in 2020 (1, 2). Men (44,120) are diagnosed twice as much as women (29,700) (2). Renal cell carcinoma (RCC) represents the major type accounting for 85% of renal cancers (2, 3). RCC has been subdivided into clear cell RCC and non-clear cell RCC histologic subtypes. Clear cell RCC (ccRCC) accounts for 75% of RCC (4). Five-years survival rate for about two third of RCC patients, those with localized disease and mainly been treated with surgery, is 93% (2). About 50% of these patients develop recurrence (5). A third of RCC patients are diagnosed with evidence of metastatic disease (2, 6). Five-years survival rate is 70% for patients with regional spread and a mere 12% for patients with distant metastases (2). Management of metastatic RCC has embraced advancements in the shape of immunomodulating, molecularly targeted and immune checkpoint inhibiting agents. These agents have improved the outcome for metastatic RCC as revealed by the 1% decrease per year in death rates from 2008 to 2017 (2, 7).

Brain metastases are developed in about 4–17% of RCC patients with about 50% of these patients are presented with multiple lesions (5, 8, 9). Untreated brain metastatic RCC patients have reported a median survival of about 3.2 months (10). Management of brain metastases from any primary site including RCC involves surgery and radiation therapy (10, 11). Surgery is mainly opted for limited brain disease (12). Multiple brain lesions are usually treated with WBRT (10, 12, 13). Though, RCC pathology has been considered radioresistant, WBRT has shown slightly improved local control (up to 60%) and median survival ranging from 3 to 7 months (9, 14, 15). SRS, on the other hand, has reported much better local control from 83 to 96% and median survival between 9.5 and 13 months (5, 16–22). Addition of WBRT to SRS have not been helpful in controlling distant brain disease (16, 23). Comparative studies have failed to report any survival advantage for combination to SRS alone (24, 25). Consequently, pattern of treatment has changed over time with more use of SRS instead of WBRT, and addition of systemic therapy has shown an improved survival for patients with RCC and brain metastases (26).

Molecularly targeted agents approved for mRCC have mainly been aimed at two targets: vascular endothelial growth factor (VEGF) associated with angiogenesis, and the mammalian target of rapamycin (mTOR), a key component in cell proliferation and known to upregulate expression of hypoxia inducible factor (HIF) (27). Since 2005, Several novel agents have been approved by the FDA for the treatment of mRCC that inhibits one of these two factors and termed as VEFGR inhibitors and mTOR inhibitors; in addition to immunotherapeutic agents (7, 27–29). VEGFR inhibitors also includes: bevacizumab, a monoclonal antibody against VEGF and the rest are termed as tyrosine kinase inhibitors (TKIs). These include: Sorafenib; sunitinib; pazopanib; axitinib; cabozatinib; lenvatinib (7, 27–29). Of these, sorafenib and sunitinib have been extensively investigated in clinic, and they have become the standard of care in patients with metastatic disease (30–32). However, their efficacy in the brain has not been determined. Nonetheless, they are associated with a decrease in incidence and development of BM in RCC patients (33–35). As well as, evidence suggests patients with RCC BM respond to these agents in the absence of local therapy (36, 37). Similarly, in a large phase III trial, temsirolimus, an mTOR (mammalian target of rapamycin pathway inhibitor) kinase inhibitor, has also shown improving OS outcome in comparison to standard of care, that has also allowed neurologically stable patients with history of surgery or radiotherapy for brain metastases (38). Moreover, combination of targeted therapy and radiation therapy was shown to be safe with conflicting reports on the efficacy front (39–42). Consequently, retrospective studies have compared efficacy outcomes in RCC BM patients treated with TKIs in combination with SRS or SRS alone (43–48). We seek to systematically review and meta-analyze these reports in order to establish a better clinical perspective for RCC patients with brain metastases.

## Methods and Materials

The PRISMA (preferred Reporting Items for Systematic Reviews and Meta-Analyses) guidelines were followed (49). A protocol of this study was registered on PROSPERO: CRD42020173796.

### Inclusion Criteria

#### Patients and Study Types

Studies comparing TKIs in combination with SRS to SRS alone for treatment of RCC patients with brain metastases. No restrictions were applied for study design.

#### Types of Interventions

Tyrosine kinase inhibitors (VEGFR tyrosine kinase inhibitors and mTOR inhibitors) in combination with SRS was termed as “Experimental group”; and the “control group” involved SRS alone.

#### Outcomes of Interest

Overall survival was the prime outcome of interest while brain control and safety outcomes were of secondary interest.

### Search Strategy

#### Databases

PubMed, EMBASE, Cochrane library, and ClinicalTrials.gov were searched until 20 March 2020. Several search terms were employed with English language restriction. Furthermore, relevant studies' references were examined for more studies.

#### Study Selection

Studies obtained were imported into Endnote X9 Software for organizing, screening, and removing the duplicates. After duplicates removal, studies were screened for title and abstracts. Studies were excluded based on the exclusion criteria. Study selection was done by two independent reviewers. Full text along with supplementary materials were obtained for selected studies. Any disagreements were resolved by the mutual consensus.

#### Data Extraction

The Cochrane Collaboration Data Collection form-RCTs and non-RCTs was used and modified for extraction of data. Information included attributes of the studies, study design, first author, country of research, publication year, number of participants, time period, and treatment regimens, main efficacy and safety outcomes for overall study group. Patient characteristics, such as age, sex, performance status (KPS), number of brain metastases, RPA classes, DS-GPA, GPA class, and MSKCC risk score. In the last, outcomes of interest were extracted. These included data on the survival, brain control and safety.

### Assessment of Risk for Bias

Risk of bias was assessed using Downs and Black checklist for assessment of the methodological quality of non-randomized interventional studies (50). The scale is composed of 27 questions covering four aspects of quality assessment. These include; reporting (10Q); external validity (3Q); internal validity (bias and confounding) (13Q); statistical power (1Q). Each question is answered yes or no or unable to determine. Score of single point is given for each yes answer except for one question in reporting section, which, carries two points, and power question, which is awarded five points. we used the modified version, as it has been used in previous studies as well, which assigns a single point to power question instead of 5 for the sack of simplification, and ambiguity avoidance (51). Gradation was assigned according to score as “excellent” (24–28 points), “good” (19–23 points), “fair” (14–18 points), or “poor” (<14 points).

### Measurement of Treatment Effect and Data Synthesis

Hazard ratios were obtained either directly from the study, or extracted from the K-M curves using the methods for incorporating summary time-to-event data into meta-analysis (52). As local control rates were also given as function of time-to-event data, a similar approach was used for extracting the hazard ratio for intracranial failure. RevMan 5.3 software was utilized for the data analysis (53, 54). Hazard ratios were pooled using inverse variance statistical method and fixed effects analysis model. *P*-value <0.05 was considered significant. Chi^2^ test was adopted for measuring the heterogeneity. The I^2^ values of 25, 50, and 75% were considered low, moderate, and high, respectively (55). If I^2^ value was >50%, random effects model was used for analysis.

## Results

**Figure 1:**
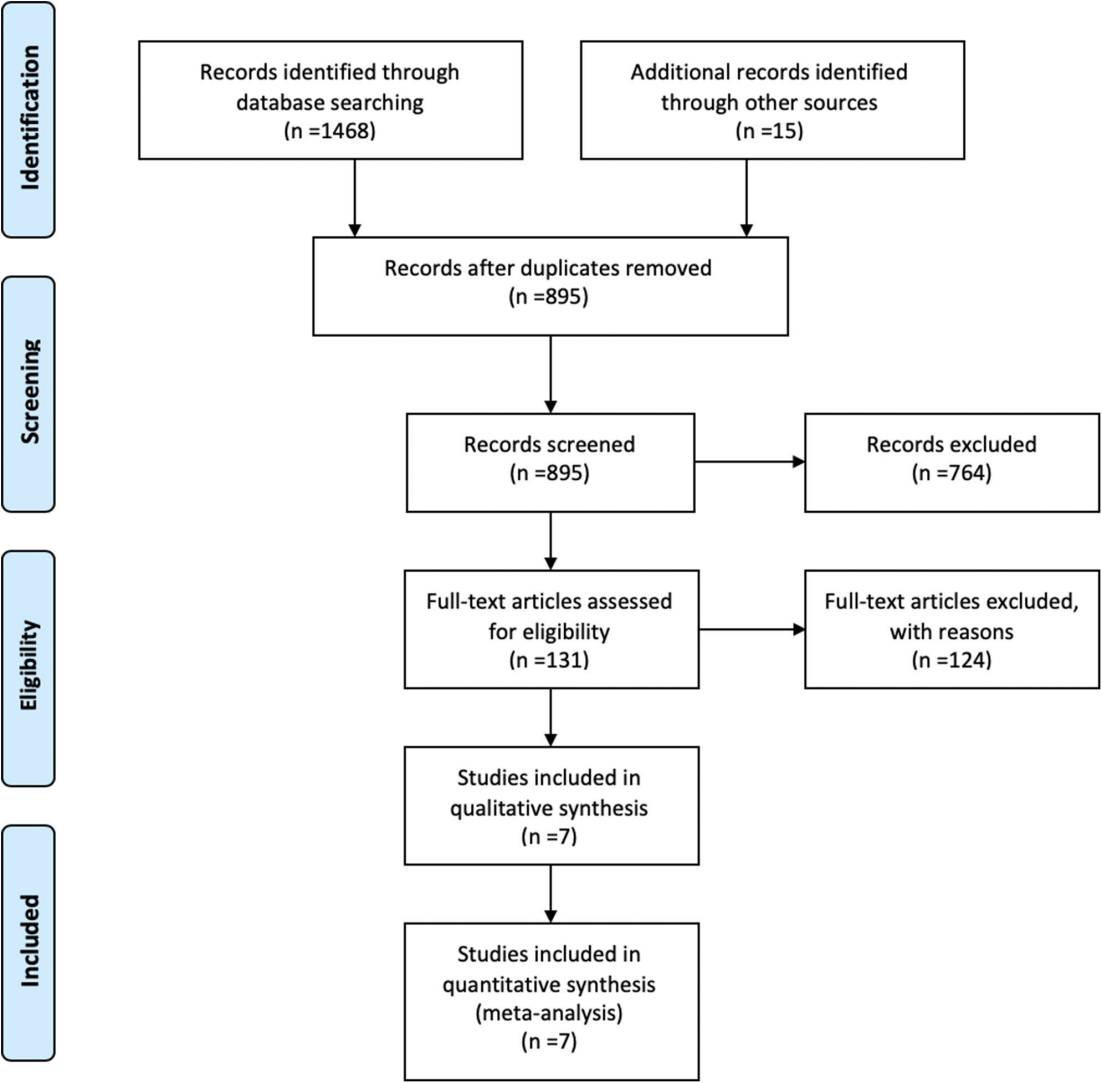
Flow diagram of study selection.

**Figure 2:**
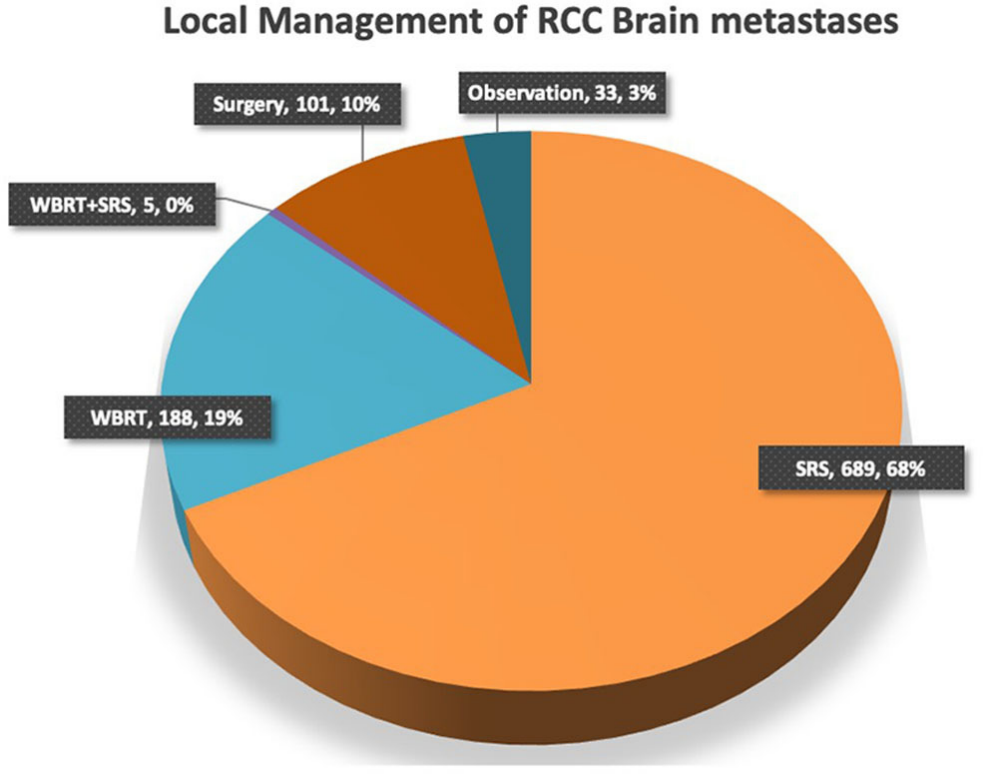
Application of SRS, WBRT, surgery or their combination in the management of RCC brain metastases.

Overall 7 studies met the inclusion criteria and were selected for meta-analysis (25, 43–48). Search strategy and selection process is demonstrated in Figure 1. These studies included a total of 897 patients; 336 in the TKIs group, and 561 in the no-TKIs group. Greater than 1,808 brain lesions were treated; either with SRS, or SRS plus TKIs (Table 1). SRS alone was applied in 68% of patients; WBRT in 19%, and surgery in 10% (Figure 2). A small number of patients had also used concurrent WBRT plus SRS (25, 47). One study also included patients managed with observation; however, numbers were balanced between non-TKI and TKIs groups (*n* = 37 vs. 38) (44). TKIs group mainly comprised of VEGFR tyrosine kinase inhibitors, and mTOR inhibitors. VEGFR-TKIs reported were: sorafenib; sunitinib; axitinib; pazopanib. mTOR inhibitors included: everolimus, and temsirolimus. Moreover, TKI group also received cytokine therapy (1%) in the study of Juloori et al.; while, immunotherapy (14%), and chemotherapy (5%) were used in the Klausner et al. study in TKI receiving patients (47, 48).

**Table 1:** General characteristics of the included studies.

**Studies**	**Period**	**Radiation**	**Targeted agents**	**No. of patients**	**Median OS**	**BPFS**	**Local failure**	**Distant failure**	**RN**	**Quality assessment**
Cochran et al. (43)	1999–2010	SRS	TKI, mTORi, bevacizumab	61	9.0 months ASR: 1-year; 38%, 2-years; 17.4%, 3-years; 8.7%		32.5 months AFFLF: 1-year; 74.3%, 2-years; 60.5%, 3-years; 40.3%	11.5 months ADFR: 1-year; 51%, 2-years; 78.6%, 3-years; 89.3%	6 patients (SRS)	19
Verma et al. (44)	2002–2007	SRS/Surgery/WBRT	Sorafenib, sunitinib	81	5.4 months (0.20–78)				4 patients (SRS)	20
Seastone et al. (45)	1996–2010	SRS	Sunitinib, Axitinib, Sorafenib	166		9.9 months (95% CI, 5.9–12.9)	AFFLF: 1-year; 75 ± 6%	12.8 months (95% CI, 8.5–21.1)	NA	15
Bates et al. (25)	2004–2013	WBRT/SRS	Sorafenib, sunitinib, pazopanib, temsirolimus	25	6.7 months (range, 2.8–22.0)	4.5 months (range, 2.5–17.3 months)			None	14
Johnson et al. (46)	2000–2013	SRS	TKI, mTORi, bevacizumab	68	–	–	–	–	NA	15
Juloori et al. (47)	1998–2015	SRS/WBRT/Surgery	TKIs mTORi cytokine (1%)	376	9.7 months		OLF: 14.9% −12-mCI: 13.4%	ODF: 24% −12-mCI: 18.6%	12-mCI; 8.0%	19
Klausner et al. (48)	2005–2015	SRS	TKIs (65%), mTORi (16%), immunotherapy (14%), chemotherapy (5%). TKIs: sunitinib (69%); axitinib (14%); sorafenib (12%); pazopanib (5%).	120	13.5 months (95% CI, 11–20) ASR: 1-year: 52%, 3-years: 29%	11 months (95% CI, 7–19)	ALCR: 1-year: 94%, 2-years: 92%	–	7%	18

*WBRT, whole brain radiation therapy; SRS, stereotactic radiosurgery; TKI, tyrosine kinase inhibitors; mTOR, mammalian target of rapamycin; OS, overall survival; ASR, actuarial survival rates; BPFS, brain progression free survival; AFFLF, actuarial freedom from local failure; OLF, overall local failure; ALCR, actuarial local control rate; ADFR, actuarial distant failure rate; ODF, overall distant failure; mCI, months cumulative incidence*.

### Characteristics of Studies

General characteristics of the studies along with quality assessment scores are outlined in Table 1. Four studies mainly involved SRS as the local therapy for treating BM (43, 45, 46, 48). Three studies, in addition to SRS, also had used surgery, and WBRT (25, 44, 47). Verma et al. study also contained patients managed with observation only (44). Bates et al. study contained very small number of patients; TKI was only used in 19% of patients in Seastone et al. study (25, 45). Quality assessment of studies ranged from 14 to 20 points of total 27 (Table 1).

### Baseline Characteristics of Patients

General characteristics of the patients are outlined in Table 2. Patients age reported in the studies ranged from median 58 to 65.7 years. Patients in the TKI group were comparatively younger in the study by Juloori et al. (59 vs. 63, *p* = 0.008) (47). Male to female ratio was observed as 3:1. It is in accordance with incidence of kidney cancer in general population as male is twice as much likely to have kidney cancer (1, 2). Imbalance was observed in the application of SRS between the groups in two studies (44, 47). Overall, 89 lesions were treated with SRS in Verma et al. study; 64 in the TKI group, and 25 in non-TKI group. Patients in TKI group in the Juloori et al. study also had received significantly more upfront SRS (81 vs. 49%, *p* < 0.001); less frequently upfront WBRT (27 vs. 55%, *p* < 0.001), and surgery (15 vs. 24%, *p* = 0.031) (47). Other characteristics; such as extent of extracranial disease, number of brain metastases, MSKCC risk score, KPS, and RPA class scores for treatment groups were reported in three studies (43, 44, 47). These characteristics were balanced in two studies; however, TKI group in Juloori et, al. study had higher KPS (90 vs. 80, *p* < 0.001), and more extracranial disease (91 vs. 82%, *p* = 0.012) (43, 44, 47).

**Table 2:** Patient characteristics and main outcomes.

**Prop**\**Studies**	**Cochran et al. (43)**	**Verma et al. (44)**	**Seastone et al. (45)**	**Bates et al. (25)**	**Johnson et al. (46)**	**Juloori et al. (47)**	**Juloori et al. (47)**	**Klausner et al. (48)**	**Meta-analysis**
Exp/Con	TKI/No-TKI	TKI/No-TKI	TKI/No-TKI	TKI/No-TKI	TKI/No-TKI	TKI/No-TKI	TKI/No-TKI	TKI/No-TKI	TKI/No-TKI
No. of patients	61 24/37	81 41/40	166 22/144	25 7/18	68 24/44	376 147/229	376 43/333	120 71/49	897 336/561
No. of lesions		216	318			912		362	<1808
Median age	62 (43–89) 60/63	59 59.2/58.6 (*p* = 0.66)	60 (31–86)	65.7 (47–83.9)		61 (31–87) 59/63 (*p* = 0.008)		58 (31–82)	
Male	50 20/30	50 24/26 (*p* = 0.75)	124	18				95	<337
Female	11 4/7	31 16/15	40	7				25	<114
SRS	61	89 64/25	166	9 2/7	68	231 119/112, *p* < 0.001		120	689
WBRT		24 14/10		11 5/6		164 39/125, *p* < 0.001			188
WBRT + SRS				5 0/5					5
Surgery		19 10/9		8		77 22/55, *p* = 0.031			101
Observation		75 38/37							33
Time of TKI induction		Before/after BM	Within 30 days of SRS	Concurrent	Within 30 days of SRS	Within 30 days of SRS	Within 30 days of SRS	37 before SRS/34 after SRS (concurrent)	
Median OS	16.6 vs. 7.2 months, *p* = 0.04	6.71 (0.29–78) vs. 4.41 (0.20–39), *p* = 0.07		7.3 (range, 4.3–58.4) vs. 4.1 (range, 1.8–22.0) HR = 0.84, *p*=0.69	21 vs. 6 months, *p* = 0.016	16.8 vs. 7.3 months, *p* < 0.001	16.4 vs. 8.7 months, *p* = 0.002		
BPFS			HR 1.13 (0.61–2.11), *p* = 0.7	HR = 1.09, *P* = 0.86					
Local control (12-mLC/CI)	93 vs. 60%, *p* = 0.01	69 and 55%, *p* = 0.051			100 vs. 88%, *p* = 0.04	11.4 vs. 14.5%, *p* = 0.11		HR 0.2 (95% CI, 0.06–0.1), *p* = 0.005	
Distant failure	HR 1.0, *p* = 0.98		HR 1.00 (0.49–2.04), *p* = 0.99		5 vs. 5 months, *p* = 0.5720	12-mCI: 16.9 vs. 10.5%, *p* = 0.003 Without upfront WBRT: 26.8 vs. 24.4%, *p* = 0.150	12-mCI: 33.3 vs. 16.7%, *p* = 0.004 Without upfront WBRT: 32.1 vs. 24.4%, *p* = 0.311		
Radiation Necrosis	6 3/3	4 2/2				12-mCI: 10.9 vs. 6.4%, *p* = 0.040	12-mCI: 15.4 vs. 7.7%, *p* = 0.20		
Neurological death	21.1 vs. 30.3%, *p* = 0.47								

*WBRT, whole brain radiation therapy; SRS, stereotactic radiosurgery; TKI, tyrosine kinase inhibitors; mTOR, mammalian target of rapamycin; OS, overall survival; BPFS, brain progression free survival; Prop, properties; Exp, experimental; Con, control; HR, hazard ratio; mCI, months cumulative incidence*.

### Meta-Analysis

#### Overall Survival

**Figure 3:**
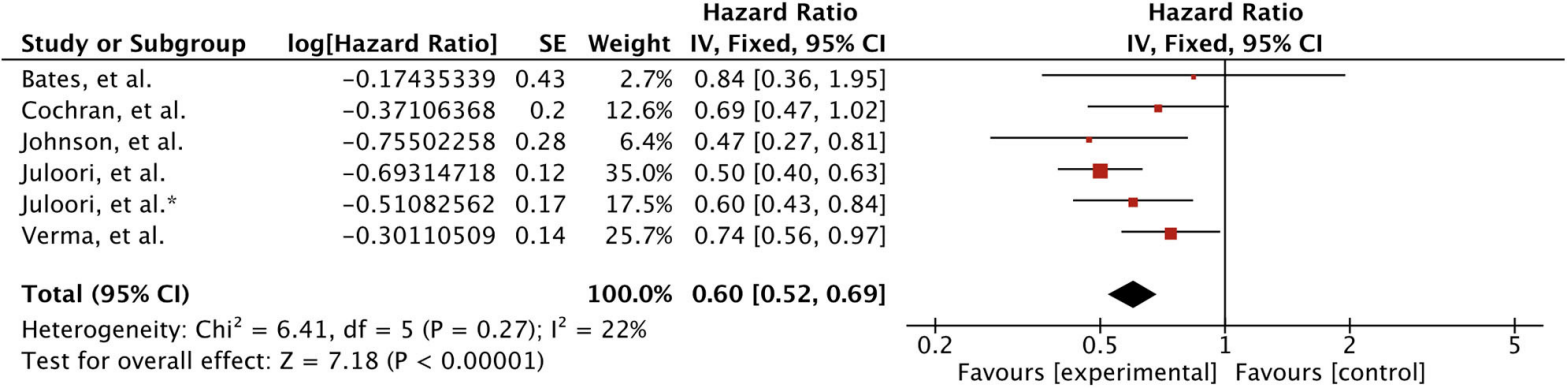
Forest plot of meta-analysis of overall survival (OS) for treatment comparison (TKIs vs. non-TKIs) in the management of brain metastases from renal cell carcinoma (RCC).

**Figure 4:**
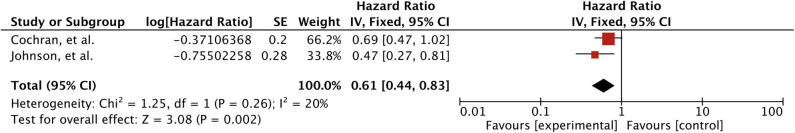
Forest plot of meta-analysis of overall survival (OS) for treatment comparison (TKIs vs. non-TKIs) involving SRS subgroup in the management of brain metastases from renal cell carcinoma (RCC).

Overall five studies reported survival outcome for treatment comparison involving 611 patients (25, 43, 44, 46, 47). Juloori et al., study reported treatment comparison for TKIs and mTORIs separately (47). Patients receiving TKIs in addition to local treatment for brain metastases were associated with significant improved survival (HR 0.60 [0.52, 0.69], *p* < 0.00001) (Figure 3). Survival remained significant when analyses were restricted to SRS only as local therapy; based on two studies (HR 0.61 [0.44, 0.83], *p* = 0.002) (43, 46) (Figure 4).

Two studies comprising 201 patients investigated effect of timing of TKIs induction in relation to BM development or SRS intervention on OS (44, 48). Verma et al. (*n* = 81) reported patients receiving TKI after BM development derived better survival in comparison to those who developed brain metastases while on TKIs, and had never used TKI (23.6 vs. 2.08 vs. 4.41 months, *p* = 0.0001) (44). Klausner et al. study (*n* = 120) revealed no systemic therapy at the time of first SRS induction for brain metastases was associated with enhanced survival (*p* < 0.0001) (48).

#### Local Tumor Control

**Figure 5:**
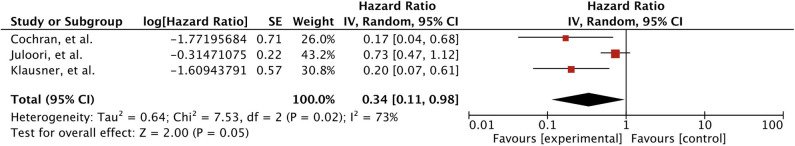
Forest plot of meta-analysis of local control (LC) for treatment comparison (TKIs vs. non-TKIs) in the management of brain metastases from renal cell carcinoma (RCC).

Overall local control rate was extracted from 3 studies involving 557 patients (43, 47, 48). There was significant better OLC rate associated with concomitant TKIs (HR 0.34 [0.11, 0.98], *p* = 0.05) (Figure 5). However, significant heterogeneity was observed (I^2^ = 73%); hence, random effects model was adopted. In addition, Verma et al. also showed better 1-year actuarial local control rates with TKI use (69 vs. 55%, *p* = 0.051) (44).

**Figure 6:**
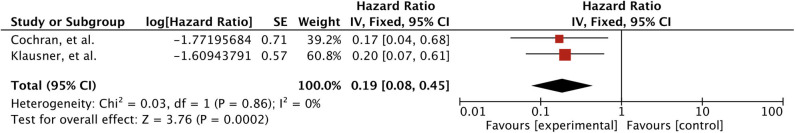
Forest plot of meta-analysis of local control (LC) for treatment comparison (TKIs vs. non-TKIs) involving SRS subgroup in the management of brain metastases from renal cell carcinoma (RCC).

When meta-analysis was restricted to studies (*n* = 181) involving SRS as the local brain treatment, significance was maintained for the treatment difference without any heterogeneity (HR 0.19 [0.08, 0.45], *p* = 0.0002) (43, 48) (Figure 6). A third study (*n* = 68) that also involved only SRS reported a significant local control (100 vs. 88%, *p* = 0.04) (46). Furthermore, subgroup analysis of BM treated with SRS (*n* = 33, 89 lesions) in the study by Verma et al. discovered a trend toward better LC with TKIs (1-year LC rates; 94.7 vs. 73.7%, *p* = 0.09) (44). Moreover, there was no significant difference among the groups according to the timing of TKI initiation with respect to BM development (after-BM vs. before-BM vs. never-TKI). One-year local control rates of 90, 53, and 74% were reported for each group, respectively (*p* = 0.18). Comparison of after-BM to never-TKI group also revealed no significant effect (*p* = 0.12) (44).

#### Distant Brain Tumor Control

**Figure 7:**
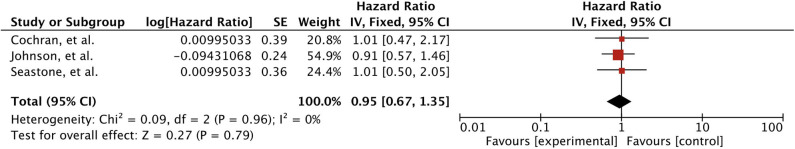
Forest plot of meta-analysis of distant control (DC) for treatment comparison (TKIs vs. non-TKIs) in the management of brain metastases from renal cell carcinoma (RCC).

There was no effect observed on distant brain control with targeted agents. Meta-analysis of the three studies (*n* = 295) involving only SRS as local treatment revealed no significant increase in the incidence of distant failure with TKIs (HR 0.95 [0.67, 1.35], *p* = 0.79) (43, 45, 46) (Figure 7). Timing of the TKI also had no effect on distant brain metastases free survival as no difference was observed between 1-year actuarial rates of DBMFS in patients receiving TKIs after (43.2%), before BM development (0%), and never receiving TKI (49%) (*p* = 0.39) (44). One study (*n* = 376) reported significant 12-months cumulative incidence of distant brain failure with TKIs (16.9 vs. 10.5%, *p* = 0.003), and mTORIs (33.3 vs. 16.7%, *p* = 0.004) use after BM (47). However, when patients receiving upfront WBRT were excluded from the analysis (even though patients receiving upfront WBRT accounted for 27%), there was no association observed between the use of TKI (26.8 vs. 24.4%, *p* = 0.150) or mTORIs (32.1 vs. 24.4%, *p* = 0.311) and distant intracranial failure (47). Verma et al. subgroup analysis also revealed a trend between previous WBRT and distant brain failure (HR 2.08, *p* = 0.17) (44).

#### Brain Progression Free Survival

**Figure 8:**
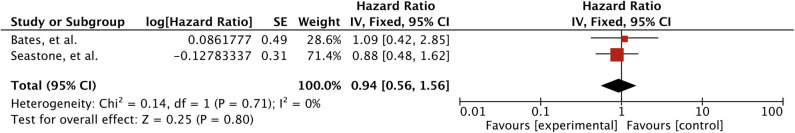
Forest plot of meta-analysis of brain progression free survival (BPFS) for treatment comparison (TKIs vs. non-TKIs) in the management of brain metastases from renal cell carcinoma (RCC).

In a small study of 25 patients, BPFS defined as time to death, local failure, or distant failure, whichever occurred first, revealed no significant difference between patients receiving TKIs or managed only with radiation therapy (HR 1.09, *p* = 0.86) (25). In Klausner et al. study, VEGF/mTOR inhibitor within 30 days before SRS was not associated with local or distant failure (HR 1.13 [0.61–2.11], *p* = 0.70) (48). Met-analysis of both reports revealed an insignificant association (HR 0.94 [0.56, 1.56], *p* = 0.80) (Figure 8).

#### Safety Profile

Overall, no systemic adverse event was reported in the studies (25, 43–48). Two studies had reported several neurologic symptoms including headache, dizziness, visual field deficits, focal weakness, intracranial edema, intracranial hypertension, intracranial hemorrhage, radiation necrosis and coma (25, 48). Other studies had mainly reported radiation necrosis for treatment difference (43, 44, 47).

#### Adverse Events

Two studies involving 145 patients reported mainly neurologic symptoms (25, 48). Klausner et al. reported Grade III/IV neurologic adverse events in 17 patients (48). RN in 8, epilepsy in 4, intraparenchymal hemorrhage in 4, severe intracranial hypertension in 6 owing to increase in symptomatic peritumoral edema, and coma in one patient that had died a week later. TKIs use was not revealed as a risk for grade III/IV or RN events on univariate or multivariate analysis. Bates et al. also revealed persistent neurological symptoms in 11 (44%) patients mainly including headache, dizziness, visual field deficits, and focal weakness (25). No significant increase in frequency of symptoms was observed between the three radiotherapeutic methods (SRS = 3; WBRT = 5; SRS + WBRT = 3, *p* = 0.65).

#### Radiation Induced Radionecrosis

One study (*n* = 376) reported significantly greater 12-months cumulative incidence of radiation necrosis in patients who had used TKIs within 30 days of SRS (10.9 vs. 6.4%, *p* = 0.04) (47). In another study, radiation necrosis was developed in a total of four patients who had undergone SRS, and had received 18–20 Gy for BM treatment. Though, patients were evenly distributed between TKI and non-TKI group, two patients in each group (44). Similarly, six patients in Cochran et al. study (*n* = 61) had experienced radiation-induced edema or necrosis after GKS, of which, three had previously been treated with targeted agents (43). Klausner et al. also revealed an overall 7% radionecrosis rate with no difference between the groups (48).

#### Neurological Death

Neurological death assessment was reported in only one study (*n* = 61) (43). Likelihood of death due to neurologic cause was 21.0% in patients receiving targeted agents (*n* = 24) and 30.3% in those not receiving targeted therapy (*n* = 37). The difference was not significant (*p* = 0.47).

### Publication Bias

**Figure 9:**
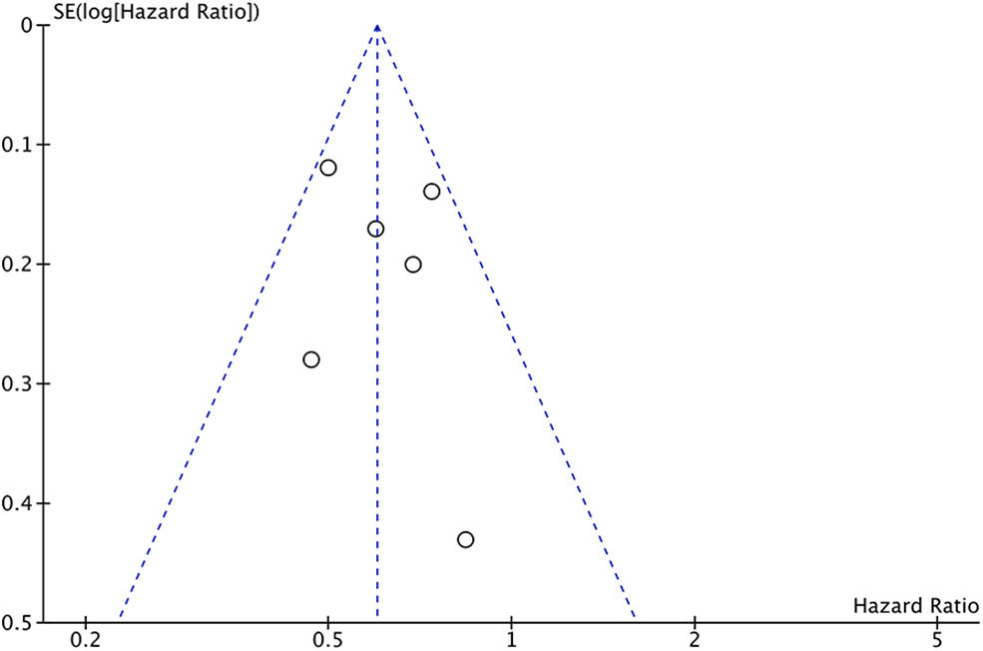
Funnel plot of publication bias assessment for overall survival.

Publication bias was assessed using the funnel plot for overall survival. All results were within the 95% CI indicating no evidence of publication bias (Figure 9).

## Discussion

Localized RCC is managed with surgery (7). Adjuvant systemic therapies include cytokine therapy with IFN-a and IL-2; and targeted therapy with VEGFR-TKI agents, and mTOR inhibitors (7, 56). VEGFR-TKIs and mTOR inhibitors have been associated with superior efficacy in terms of PFS, OS, and ORR, for the treatment of advanced RCC in comparison to placebo or INF-a (56). Renal cell carcinoma is one of the histology that metastasize to brain in advanced stage disease, and is associated with poor survival (57). Radiation therapy is required for treating brain metastases, and selection of proper strategy is based upon several prognostic factors (57). Nonetheless, SRS has been used widely as the local treatment of choice for brain metastases in renal cell carcinoma due to radioresistance to WBRT (26). Preclinical evidence lends support to the idea of combining targeted therapy and radiation therapy; as *in vitro* and *in vivo*, synergism of kinase inhibitors and ionizing radiation have been demonstrated in several cancer cell lines (58–62). Various clinical studies involving patients with RCC BM have also reported better intracranial responses even in the absence of local therapy (33–42). Hence, several studies have compared the era before and after targeted therapy have entered the clinic for the treatment of RCC brain metastases in terms of efficacy and safety outcomes (25, 43–48).

Result of this study revealed an improved overall survival for RCC brain metastatic patients receiving TKIs. Survival advantage remained significant even when analysis was restricted to studies involving SRS as the only local treatment. Whether this surge of survival in EGFR-TK/mTORi era is from the efficacy of these agents in brain could be queried. Although, there is no biological mechanism to describe the survival benefit, several *in vitro* and *in vivo* studies have shown synergism between radiation and targeted agents (58–69). Radiation promote angiogenesis through increasing the expression of VEGF and several other angiogenic factors, such as angiotensin-1/2, and Tier-1 (receptor for Ang-1/2); as well as growth factors, such as TGF-a, MAPK) (63–66). Low dose ionizing RT was shown to promote tumor growth and metastasis through activation of VEGFR2 and its upregulation have been associated with radioresistance (64, 67). While targeted agents have shown to increase radiosensitivity of tumors through several mechanisms. Sorafenib sensitizes tumor to radiation through inhibition of radiation-induced VEGFR2 and its downstream ERK signaling pathway; induces DNA damage, and block/delay DNA repair (58–61). Sunitinib has also shown to increase radiosensitivity of both tumor and endothelial cells in preclinical studies (62, 68). Temsirolimus, through induction of autophagy, had increased *in vitro* radiosensitization of RCC (69). Sorafenib, sunitinib, pazopanib, and cabozatinib, all have demonstrated safety and brain responses in several case reports and trials (70–78). Furthermore, significantly better control reported in some of these studies as well as the result of our study suggest their efficacy in the brain as well (43, 44, 46, 48). Counterarguments include preclinical evidence suggesting restriction of sorafenib and sunitinib transport into the brain (35). Moreover, their use was also associated with delaying the development of BM in a retrospective analysis which may suggest a better systemic disease control with these agents leading to a better survival eventually (33, 35).

Overall local control results favored TKIs use. However, heterogeneity was high. We analyzed the result with exclusion of study involving WBRT or surgery use, TKIs were associated with better local control without any heterogeneity involving the studies that had used SRS only. Juloori et al. study comprising 376 patients, reported no local control advantage for TKI use (47). Significant SRS use in TKI group; and WBRT and surgery in the no-TKI group, might have balanced the odds as WBRT plus SRS is associated with significantly better local control in the treatment of BM (79). Similarly, surgery has also been reported to have equal effectiveness to SRS alone in treating brain metastases (80). This could also be reflected in the result of distant intracranial failure as TKI use was reported with greater risk for failure unless comparison was done with exclusion of patients receiving WBRT use (47).

Timing of kinase inhibitor induction in relation to BM development and SRS intervention have been argued for clinical efficacy (44, 48, 81–83). Two retrospective studies investigating relationship of BM development and survival for RCC patients revealed Metachronous BM development was associated with better OS as compared to synchronous BM (81, 82). OS from initial RCC or mRCC diagnosis was better for metachronous BM patients treated with either local treatment (SRS, WBRT, surgery) or systemic therapy alone (81, 82). However, there was no difference in OS from BM diagnosis for both groups (81, 82). A multicenter retrospective study investigating timing of targeted therapy induction for mRCC patients with BM also reported synchronous BM trended toward poor survival from commencement of targeted therapy (*p* = 0.06) (83). Nonetheless, metachronous BM while on targeted therapy demonstrated worst survival outcome in comparison to both synchronous BM group, and metachronous BM group that received targeted therapy after BM development (*p* < 0.001) (83). A similar observation was reported by Verma et al. (44) In his study, significant improvement of median survival was reported for patients receiving TKI post-BM development (23.6 months) in comparison to pre-BM group (2.08 months) (*p* = 0.0001) (44). Similarly, no systemic therapy at the time of first SRS was also identified as a factor associated with better survival on univariate and multivariate analysis in the study by Klausner et al. (*p* < 0.0001) (48). It can be hypothesized that patients receiving systemic therapy before BM development may reflect a late stage disease; as BM development could be delayed with sorafenib in RCC patients (33, 35). On the other hand, BM development while on TKI may also represent a changed tumor biology or adaptive resistance to TKI. This could be further supported as naïve group trended better in local (90 vs. 53%) and distant brain control (43.2 vs. 0%) compared to progression group in the study of Verma et al. (44). These observations suggest BM development while on TKI may represent a different entity and require special consideration.

According to these reports, combination therapy is safe as compared to SRS alone (25, 43–48). No systemic adverse events were reported in these studies (25, 43–48). Adverse events associated with sorafenib and sunitinib alone are reported elsewhere in literature. Fatigue and diarrhea were the most common systemic adverse events associated with both sorafenib and sunitinib in clinical trial studies (30, 32). While sorafenib was further revealed to cause cardiovascular (hypertension and cardiac ischemia) and skin toxicities (rash and hand-foot skin reactions) (30). Sorafenib was also associated with grade II neurologic (sensory neuropathies). However, there was no case of CNS adverse reactions revealed in these studies (30, 32). It may imply that without SRS, these agents are less likely to penetrate CNS (BBB) in high enough doses to cause side effects. Though, in our analysis, two studies reported several neurologic side effects, but these were not related to systemic therapy (25, 48). Therefore, these neurologic symptoms may only be due to SRS use alone. Adding these agents may not increase the odds of such neurologic symptoms.

RCC patients receiving SRS for brain metastases are at double odds for developing radionecrosis as RCC histology embodies an independent risk factor for RN, while RN signifies a dose-limiting toxicity associated with intracranial SRS (84, 85). Four studies involving 287 patients (minimum 25, maximum 120) indicated no increase in RN with addition of TKIs to SRS. Since reported 1-year cumulative incidence of RN is 5–10%, these studies appear to be not powered enough to detect such an association, particularly if consider the number of patients receiving TKIs within 30 days of SRS (86, 87). As Staehler et al. also had failed to demonstrate any incidence of radiation necrosis with concomitant SRS and VEGFR TKIs in the treatment of 51 RCC BM patients (39). On the contrary, one study, by far the largest, comprising 376 patients reported significant increase in 12-months cumulative incidence of RN with use of TKIs within 30 days SRS but not mTORIs (47). In retrospective study by Kim et al., had observed increased 12-months cumulative incidence within the patients receiving concurrent VEGFR TKIs and SRS (14.3 vs. 6.6%, *p* = 0.04) (88). When analyzed in subgroups by concurrent WBRT induction with SRS status, patients receiving concurrent WBRT were associated with significant incidence of RN (21.4 vs. 0.0%, *p* = 0.03) as compared to without (13.1 vs. 8.3%, *p* = 0.11). Signifying the role of WBRT given concurrently with SRS in causing RN. Juloori et al. study also included patients who were treated with WBRT in addition to SRS but subgroup analyses were not reported (47). In addition, Kim et al. further identified KPS and extracranial metastases associated with RN (88). Both of these factors were also significantly different between the treatment groups in Juloori et al. study (47).

Results of this study is limited by several factors. First of all, all the included were of retrospective nature spanning over a large duration of time. Retrospective studies are prone to selection bias, recall bias or misclassification bias and are subject to confounding (89). One study had very small number of patients (*n* = 25) (25). Only 19% of patients had received TKI in the study by Seastone et al. (45). An imbalance of local therapy (SRS, WBRT, surgery) was observed between TKI and non-TKI groups in Juloori et al. study (47).

## Future Perspective

All the studies included in our analysis mainly involve the use of sorafenib and sunitinib (25, 43–48). There are newer agents entering the therapeutic spectrum of metastatic melanoma (29). Pazopanib was shown to be as efficacious as sunitinib in treating metastatic melanoma, and have also demonstrated intracranial activity in these patients in several case reports (76, 77, 90–92). A recently approved agent, cabozatinib, has shown superior efficacy compared to sunitinib in a phase 3 trial (93, 94). Cabozatinib was able to induce brain complete response prior to radiation therapy in mRCC (78). A recent retrospective study comprising 12 RCC brain metastatic patients with at least one prior anti-angiogenic therapy revealed an ORR of 50% with disease control rate of 75%. In all 5 patients that had been treated for both systemic and brain-directed approach combinedly had obtained intracranial disease control without increase toxicity (95). Immunotherapeutic agents, all three types of agents anti-CTLA-4, anti-PD-1, and anti-PD-L1, have been assessed for efficacy in mRCC patients (96, 97). Combination of nivolumab (anti-PD-1) and ipilimumab (anti-CTLA-4) were shown to have superior efficacy compared to sunitinib (96). A similar better activity was observed for combination of atezolizumab (anti-PD-L1) and bevacizumab against sunitinib as well (97). Though, brain metastatic patients were not included in these trials, improved outcomes promise a better result for brain metastatic patients as it has been demonstrated in the case of melanoma brain metastases (98). Immunotherapy in MBM patients were revealed to operate synergistically with SRS, and had obtained significantly better survival in comparison to SRS alone as well as a trend toward numerical superiority to BRAF inhibitors was seen (98–100). Synergism of immunotherapy and radiation therapy is revealed to have abscopal effect which is defined as the activity of the combination in non-irradiated area (99, 101–103). Similarly, absence of immunotherapy after radiosurgery was demonstrated to be associated with a decrease in developing of new brain metastases (104). In this direction, a recently concluded phase II trial (NIVOREN) have compared two treatment cohorts receiving nivolumab for treating RCC brain metastases (105). Cohort A included RCC brain metastatic patients without previous irradiation therapy for BM while cohort B comprised of RCC BM patients with previous radiation therapy mainly SRS (85%). A comparatively better intracranial progression free survival was observed in cohort B despite more negative prognostic factors (performance status, IMDC risk, single brain metastasis, smaller brain lesions, tumor grade ≤2) compared to cohort A. At 6-months PFS was 49.4% (95% CI: 31.7–64.8%) in cohort B compared to 23.8% (95% CI: 11.1–39.2%) in cohort A. Similarly, median PFS was 2.7 months (95% CI; 2.3–4.6) in cohort A compared to 4.8 months (95% CI: 3.0–8.0) in cohort B. Prior radiotherapy was identified on multivariate analysis to decrease the risk for intracranial PFS in cohort B by half (HR 0.49 (95% CI: 0.26–0.92) (105). Furthermore, as mentioned earlier, sorafenib and sunitinib access to the brain was restricted by P-glycoprotein (ABCB1) and breast cancer resistance protein (ABCG2) (35, 106). Oral elacridar (a dual ABCB1/ABCG2 inhibitor) and sunitinib coadministration was shown to increase the accumulation of sunitinib in the brain suggesting this combination could further enhance the activity of these agents in the brain (107). These data are encouraging and suggest an emerging dimension for RCC brain metastases with more efficacious targeted agents, and immune checkpoint inhibitors is on the rise.

## Conclusions

Our results indicate that survival is boosted without any increase in intracranial toxicity in patients receiving TKIs in addition to local radiation treatment for RCC BM. Local control augmentation suggests improved survival might be a result of TKIs/SRS synergism. Timing of TKIs induction was observed to be crucial with respect to BM development and SRS in deriving survival and avoiding radiation necrosis. Randomized controlled trials are merited to validate survival outcome and investigate various aspects around the use of TKIs in combination with SRS.

## Data Availability Statement

All datasets generated for this study are included in the article/supplementary material.

## Author Contributions

All authors listed have made a substantial, direct and intellectual contribution to the work, and approved it for publication.

## Conflict of Interest

The authors declare that the research was conducted in the absence of any commercial or financial relationships that could be construed as a potential conflict of interest.
